# Applying Flax FRP in an Innovative Closed-Shape Stirrup for Concrete Beams

**DOI:** 10.3390/ma15082927

**Published:** 2022-04-17

**Authors:** Cheng Chen, Caiwei Li, Yingwu Zhou, Lili Sui, Xue Li

**Affiliations:** 1 Guangdong Provincial Key Laboratory of Durability for Marine Civil Engineering, Shenzhen University, Shenzhen 518060, China; mnmchen@szu.edu.cn (C.C.); 1910472061@email.szu.edu.cn (C.L.); ywzhou@szu.edu.cn (Y.Z.); suill@szu.edu.cn (L.S.); 2 Shenzhen Institute of Information Technology, School of Traffic and Environment, Shenzhen 518172, China

**Keywords:** fiber-reinforced polymer (FRP), shear performance, reinforced concrete, life cycle analysis (LCA)

## Abstract

Under the background of climate change, the steel industry is considered one of the least eco-friendly industries. Flax fiber-reinforced polymer (FFRP) is an emerging sustainable alternative to steel reinforcement bar; however, its application is much restricted due to its interior material properties. This paper proposed a novel way to form closed-shape stirrups with FFRP, which is suitable for replacing steel stirrups. A multi-disciplinary investigation was conducted concerning the structural and environmental performance of FFRP stirrups in reinforced concrete (RC) beams. Seven specimens were tested under a three-point bending load. The FFRP stirrups substantially increased the shear capacity and ultimate vertical displacement by 77% and 74%, respectively, and shifted brittle failure to ductile failure. The closed-shape stirrups avoided the stress concentration and increased the utilization of FFRP tensile capacity to over 80%. Decreasing the spacing of FFRP stirrups effectively increased the shear capacity and ductility; increasing the width or layer of FFRP stirrups improved ductility only. A life cycle assessment (LCA) was later performed to evaluate and compare the environmental performance of steel, FFRP, and carbon FRP stirrups. As compared to carbon FRP and steel ones, FFRP stirrups substantially decreased the global warming and fossil depletion potential by over 60%. The main contributors to the environmental impacts of FFRP stirrups were the heavy metal released into the water and terrestrial environment during the cultivation process.

## 1. Introduction

In reinforced concrete (RC) structures, fiber-reinforced polymer (FRP) is a promising alternative to steel reinforcement bar due to its comprehensive advantages in terms of high strength, low density, and resistance to corrosion [1]. The high strength-to-weight ratio of FRP reduces the self weight [2], and the resistance to corrosion solves the durability problems in off-shore RC structures [3]. The elastic modulus of FRP, however, is lower than that of steel reinforcement. Most design codes [4,5] impose a deformation limit on RC structures upon the serviceability state, which is often exceeded when FRP is used as tensile reinforcement. On the other hand, stirrups are closest to the concrete surface and prone to environmental corrosion, and the resistance to corrosion makes FRP an ideal material for the stirrups in RC structures.

However, the wide applications of FRP stirrups in the construction industry is limited by various reasons. Traditional FRP composites use epoxy resin to impregnate synthetic—such as carbon, glass, and basalt—fibers to form FRP. The synthetic fiber is responsible for resisting the tensile force and the epoxy resin bundles’ individual fibers together. However, the elastic modulus of synthetic fiber is much higher than that of the concrete substrate, and the resultant incompatibility often leads to interfacial debonding between FRP and concrete [6,7]. Though various forms of anchorages have been proposed [8,9,10] to improve the composite action between FRP and concrete, the utilization of FRP composites remains low, and only a small portion of FRP’s tensile capacity is permitted to be used as an effective strain of FRP by mainstream design codes [11,12,13]. The production of synthetic fiber originates from the extraction of crude oil and requires multiple sophisticated processes before the synthetic fiber is produced, and the material cost of synthetic fiber is high. An existing estimate [14] showed that the replacement of steel reinforcement bar with FRP ones yields a 99% increase in initial material cost. Another drawback of synthetic FRP is its environmental performance. Significant amounts of pollutants and greenhouse gases (GHG) are emitted to the environment during the manufacturing process of FRP. In summary, the compatibility issue, poor environmental performance, and high cost of synthetic FRP impede its wide applications.

In recent years, natural FRP has emerged as a new generation of FRP due to its balanced performance in terms of material utilization, environmental impact, and material cost [15,16]. Natural FRP uses renewable materials of satisfactory material properties instead of synthetic fiber to form FRP. As compared to steel or synthetic FRP, natural FRP is more flexible to form reinforcement in different shapes and eco-friendlier with lower tensile strength. For instance, flax fiber has a tensile strength ranging from 300 to 1000 MPa [17], and an elastic modulus within 20–100 GPa [18]. Natural FRP has an elastic modulus close to that of concrete, yielding high material utilization. In recent studies applying natural FRP for structural retrofitting [19,20], most natural FRP ruptured without anchorage, meaning the corresponding tensile strength was fully utilized. According to several life cycle assessments and material cost evaluations [21,22], furthermore, natural FRP was proved to be eco-friendly and cost-efficient. In the combat against global climate change, replacing steel stirrups with eco-friendly natural FRP stirrups reduced the usage of steel/synthetic FRP stirrups, and the corresponding environmental impact. In the aspect of material cost [19], jute FRP—when used for structural retrofitting—costed 40% lower than synthetic FRP to achieve a unit increase in load-capacity. Although natural FRP has great potential for structural application, very limited research has been conducted concerning its performance when used as stirrups in RC beams. Recent research [23] revealed that flax FRP (FFRP) stirrups, fabricated using the hand wrapping method, could contribute a shear capacity similar to steel stirrups. However, the utilization of FFRP was low due to the debonding between FFRP and concrete, and the excessive amount of epoxy resin increased both material cost and environmental impacts.

In light of the need for a thorough investigation, this paper conducts a multi-disciplinary assessment of FFRP stirrups in terms of shear behavior and environmental impact. An innovative closed-shape stirrup was adopted to fabricate FFRP stirrups and fully utilize the tensile capacity. The FFRP stirrups were used for shear reinforcement in a total of seven specimens. The effects due to the width, spacing, and layer of FFRP stirrups were investigated. The environmental impact of FFRP stirrups was then calculated using a life cycle assessment (LCA) and compared to those of steel and carbon FRP (CFRP) stirrups. A normalization approach-based LCA was proposed and adopted to consider the vast variance in material properties and usage. The LCA results casted insights into the environmental performance of FFRP, as compared to steel reinforcement and synthetic FRP, on the structural level. The multi-disciplinary assessment in this study confirmed the great potential of closed-shape stirrups using FFRP as an eco-friendly alternative to traditional bent stirrups of steel reinforcement and synthetic FRP.

## 2. Experimental Program

### 2.1. Specimen

**Figure 1:**
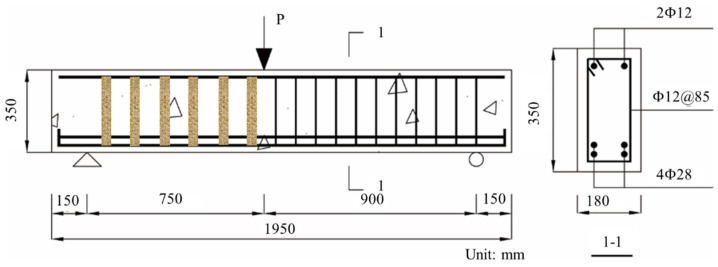
Specimen configuration.

The experimental program was comprised of seven specimens, whose configuration is shown in Figure 1. Each specimen was 180 mm wide, 350 mm high, and 1950 mm long. The distance between supports was 1650 mm, which was divided by a concentrated load into a 900 mm-long reference span and a 750 mm-long test span. Each specimen had two 12 mm-diameter steel reinforcement bars as compressive reinforcement, and four 28 mm-diameter steel reinforcement bars as tensile reinforcement. A steel stirrup of 12 m diameter was placed at intervals of 85 mm within the reference span and no steel stirrup was used within the test span. The test specimen was designed to be over-reinforced to facilitate the calculation of shear capacity, and the corresponding reinforcement ratio was 3.9%. The flexural capacity of the over-reinforced beam is usually 2–3 times of shear capacity, and the shear capacity can thus be reached first even with the FFRP stirrup, which enables the analysis of the overall shear capacity and the component contribution from zero to shear failure. The shear reinforcement ratio within the reference span was 4.6%, avoiding shear failure within the reference span.

**Figure 2:**
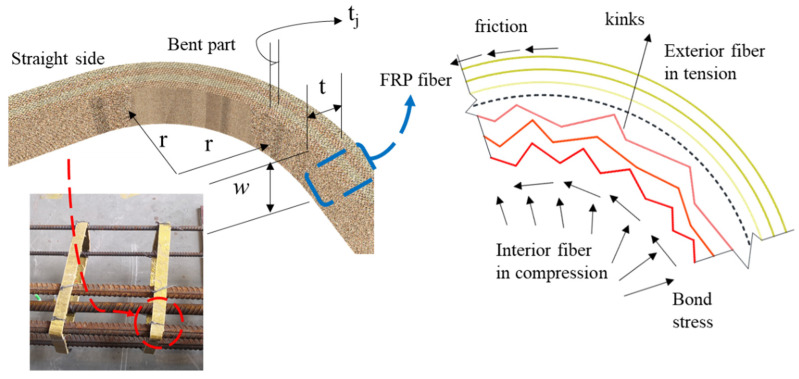
Stress concentration of bent FRP.

**Figure 3:**
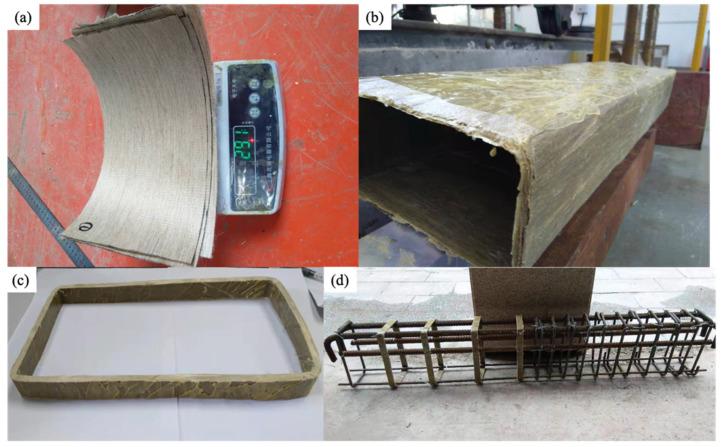
FFRP stirrups: (**a**) flax fabric; (**b**) flax FRP tube; (**c**) FFRP stirrup; and (**d**) steel cage.

A novel closed-shape stirrup was used in this study. Conventional FRP stirrups were bent using straight FRP bars, and during the bending process, the fibers on the interior side (within the neutral axis, shown by dashed line in Figure 2) were subjected to compressive stress and kinked, whereas the fibers on the exterior side were tensioned [24], both lowering the effective strength at the bent parts. Existing studies showed [25] that most FRP stirrups failed at the bent parts, and the tensile strength of FRP at the bent parts was around 40% lower than that of the straight side. Furthermore, the bent FRP stirrups were found [26] to slip from concrete despite the use of the overlapping zone. The contribution of the FRP stirrup to shear capacity was hence limited. To reduce the initial stress at the bent parts of the FRP stirrup, the FFRP stirrup in this study adopted the wet lay-up method. The flax fabric was first rinsed to remove the weak substance and dried at 50 °C for at least 24 h. The dry fabric (Figure 3a) was then impregnated with epoxy resin, wrapped around a rectangular form, and left to cure for seven days. The FFRP tube was detached from the form (Figure 3b) and cut into designated dimensions (Figure 3c). The cut flax FRP stirrups were then used to tie the steel reinforcement bar and to form a steel cage for the specimen (Figure 3d). During the wet lay-up process, the dry flax fabric was still soft and free from any stress, and the initial stress level of the stirrup was hence reduced.

The seven specimens in this experimental program varied in the FFRP stirrup within the test span. One specimen used no shear reinforcement within the test span and was referred to as the reference specimen. The FFRP stirrups were used in the other six specimens. The details of flax FRP stirrups are summarized in Table 1 for all specimens. Three design parameters were considered in this paper: layer, width, and spacing of FFRP stirrups. The nomenclature of the specimens in this paper is as follows: flax layer × FFRP width @ FFRP spacing. For instance, specimen 10 × 60 @ 100 used ten-layer 60 mm-wide flax FRP stirrups spaced at 100 mm within the test span. Based on an existing study in the literature [27], the studied ratio of shear reinforcement was within 1% to 5% (Table 1).

### 2.2. Materials

**Figure 4:**
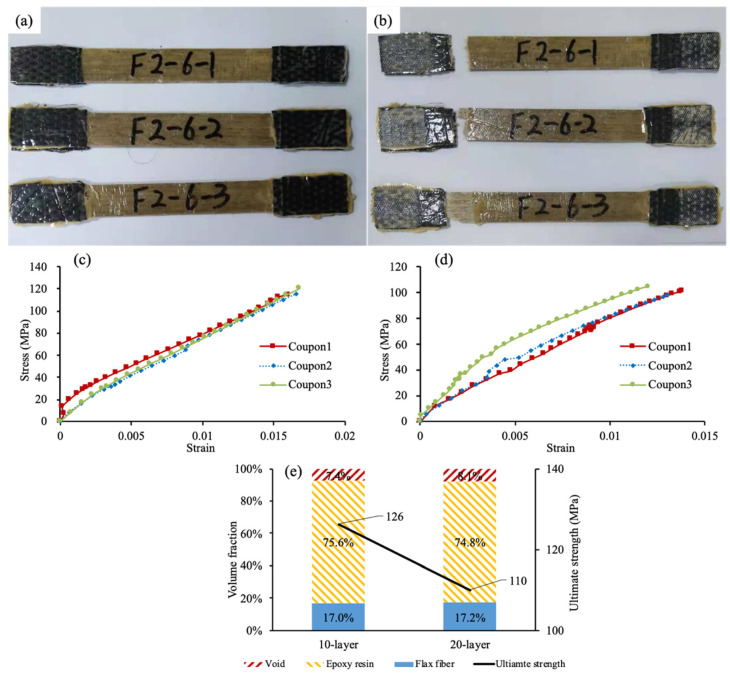
FFRP material properties: (**a**) coupon specimen; (**b**) coupon rupture; (**c**) stress–strain relation (10-layer); (**d**) stress–strain relation (20-layer); and (**e**) summary.

Flax fabric was used to fabricate a flax FRP coupon in Figure 4a using the wet lay-up method as specified in ISO 527-4 [28]. Three identical flax FRP coupons were fabricated and left to cure under room conditions for at least seven days. The flax FRP coupons were loaded under uniaxial tension until FRP rupture (Figure 4b). The average constitutive relation of flax FRP is presented in Figure 4c,d. The high stress level of coupon 3 in Figure 4d was possibly due to local FRP failure outside the gauging zone. The weights of dry flax fiber and FFRP coupons were measured by scale, and the weight of epoxy resin can thereby be determined by subtracting the weight of dry flax fiber from that of the FFRP coupon (Equation (1)):

(1) $$w_{FFRP}-w_{fiber}=w_{epoxy}$$

where *w_FFRP_*, *w_fiber_*, *w_epoxy_* = weight of FFRP, flax fiber, and epoxy resin, respectively.

The volumes of flax fiber and epoxy resin can be determined using the respective density, and the volume of voids in the FFRP coupon is the difference between the total volume and the sum of flax fiber volume and epoxy resin volume:

(2) $$V_{fiber}=\frac{w_{fiber}}{\rho_{fiber}}$$

(3) $$V_{fiber}=\frac{w_{fiber}}{\rho_{fiber}}$$

(4) $$V_{void}=V_{FFRP}-V_{fiber}-V_{epoxy}$$

where *V_FFRP_*, *V_fiber_*, *V_epoxy_* = volume of FFRP, flax fiber, and epoxy resin, respectively; *ρ_fiber_*, *ρ_epoxy_* = density of flax fiber and epoxy resin, respectively. Therefore, the volume fractions of flax fiber, epoxy resin, and voids are determined by normalizing the respective volume using total volume:

(5) $$v_{fiber}=\frac{V_{fiber}}{V_{FFRP}}$$

(6) $$v_{epoxy}=\frac{V_{epoxy}}{V_{FFRP}}$$

(7) $$v_{void}=\frac{V_{void}}{V_{FFRP}}$$

where *v_FFRP_*, *v_fiber_*, *v_epoxy_* = volume fraction of FFRP, flax fiber, and epoxy resin, respectively. The tensile strength of the FFRP coupon can be obtained by dividing the rupture force of the FFRP coupon by the sectional area of flax fiber and epoxy resin:

(8) $$f_{FFRP}=\frac{F_{FFRP}}{b_{FFRP}t_{FFRP}\left(1-v_{void}\right)}$$

where *f_FFRP_* = tensile strength of FFRP; *b_FFRP_*, *t_FFRP_* = width and thickness of FFRP, respectively. The ultimate strength was obtained as 126 MPa and 110 MPa for the 10-layer and 20-layer FFRP, respectively, and the corresponding elastic moduli were 7.4 GPa and 7.9 GPa. According to the manufacturer, the tensile strengths of flax fiber and epoxy resin were 500 MPa and 39.9 MPa, respectively, and the corresponding elastic moduli were 30 GPa and 2.4 GPa, respectively.

As per the Chinese design code for concrete structures [29,30], the compressive strength of concrete was obtained, using three concrete cubes of 150 mm × 150 mm × 150 mm, as 39.1 MPa for the 28-day and 41.3 MPa for the test day. Three steel reinforcement bars of 6 mm, 12 mm, and 28 mm diameter were used in this study, whose material properties of the steel reinforcement bar were obtained following the ASTM A955/A955M19 [31] code. The yielding strength of 6 mm, 12 mm, and 28 mm-diameter steel reinforcement bars was 482 MPa, 451 MPa, and 450 MPa, respectively. The corresponding ultimate strength was 551 MPa, 620 MPa, and 617 MPa, and the elastic modulus was 238 GPa, 192 GPa, and 229 GPa, respectively. All material properties are listed in Table 2 for summary.

### 2.3. Test Setup

**Figure 5:**
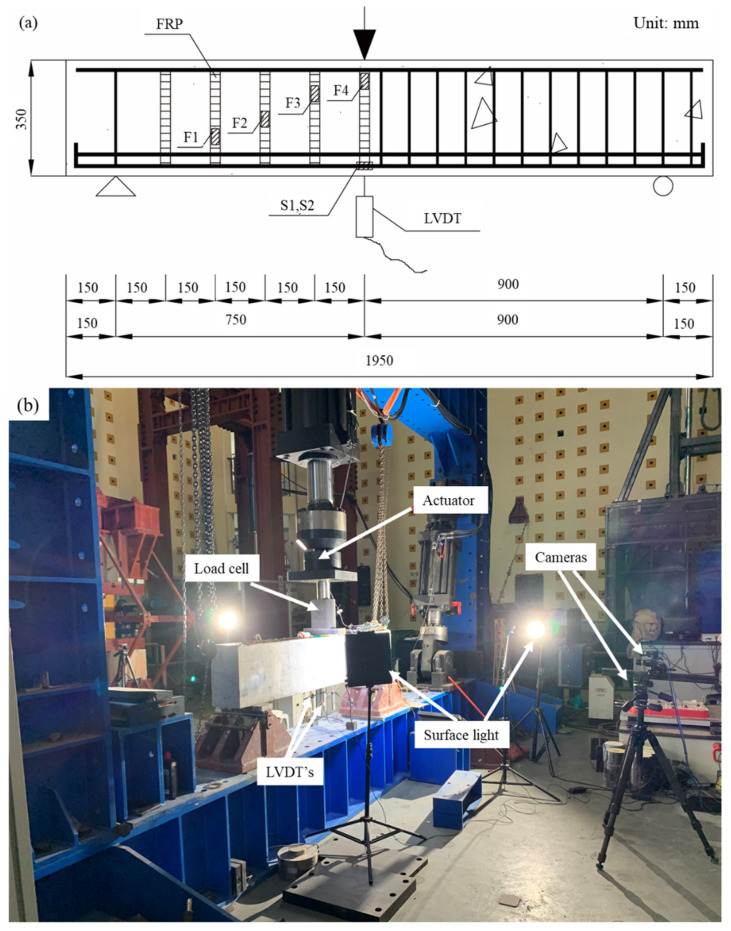
Instrumentation: (**a**) strain gauges and LVDT; and (**b**) DIC system.

As shown in Figure 5, the concentrate load was applied by a 100,000 kN hydraulic loading machine at a rate of 0.2 mm/min. Strain gauges—denoted by F1 and F2—were bonded to a flax FRP stirrup (Figure 5a), forming a diagonal line connecting the support and the loading point. Two strain gauges (S1 and S2) were used to monitor the tensile strain of steel reinforcement bar on a lower level under the loading point. Two linear variable displacement transducers (LVDTs) were installed under the loading point to measure the vertical displacement. All strain gauges, load cells, and LVDTs were wired to a DEWE-Soft data acquisition system for sampling at a reading per second.

A load cell was placed between the actuator and the specimen to monitor the load level, and the strain and cracks on the surface of concrete were monitored using a digital image correlation (DIC) system (Figure 5b). The DIC system—comprised of two cameras, two surface lights, and processing software—is a nondestructive system to track the movement of speckles on concrete. Based on the theorem of elasticity, the displacement field within the speckled plane was then used for obtaining the strain field and crack dimensions.

## 3. Shear Performance

### 3.1. Test Results

**Figure 6:**
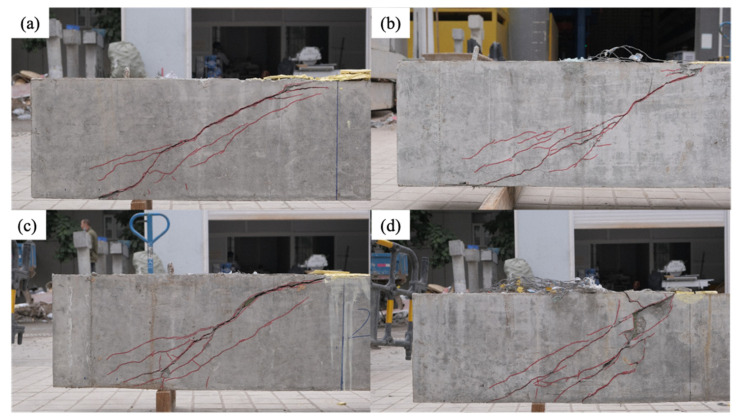
Failure mode for specimen: (**a**) reference; (**b**) 20 × 30 @ 200; (**c**) 20 × 30 @ 150; and (**d**) 20 × 30 @ 100.

Table 3 lists the load and vertical displacement upon the yielding state, peak state, and ultimate state, and the inclination angle of the major shear crack. The yielding point was determined using the bilinear method specified in [32]. The reference specimen failed by shear, and all RC beams using FFRP stirrups failed by the rupture of FFRP stirrups followed by shear failure. Figure 6 shows the typical failure mode. The major shear crack initially formed at mid-height of the center of the test span, and propagated toward the support and loading point during the loading process. The widening of the major shear crack tensioned the embedded FFRP stirrups until rupture. At peak load, a significant decrease in load capacity was accompanied by a loud sound of FFRP rupturing. After the FFRP rupture, the concrete and remaining FFRP stirrups were unable to resist the applied load, leading to shear failure in concrete and subsequent rupture in other FFRP stirrups. As the ratio of shear reinforcement increased, the crack pattern shifted from a single major crack (Figure 6a,b) to distributed shear cracks (Figure 6c,d), which were also referred to as the crack band. In those specimens with a high ratio of shear reinforcement (Figure 6d), the concrete cover detached from FFRP stirrups upon ultimate failure, and part of the FFRP stirrups were exposed.

### 3.2. Load–Vertical Displacement Relation

**Figure 7:**
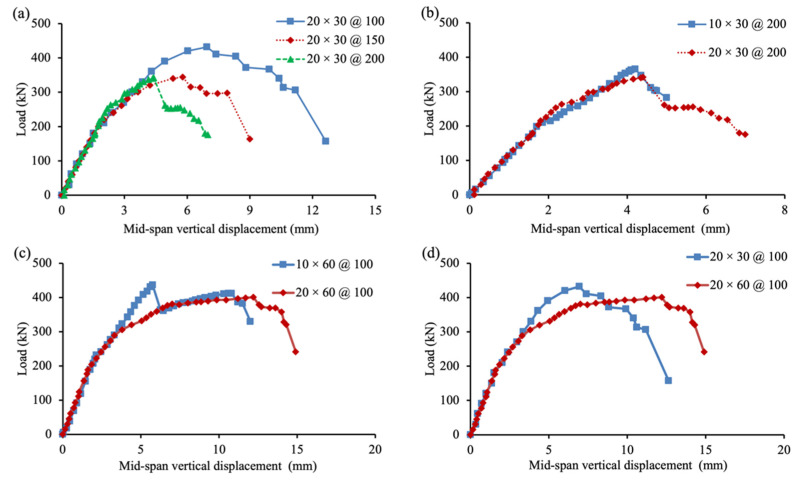
Load–vertical displacement relation. (**a**) Spacing comparison; (**b**) Tickness comparison; (**c**) Tickness comparison; (**d**) Width comparison.

Figure 7 shows the load–vertical displacement relations for all seven specimens in this experimental program. The effect due to the spacing of FFRP stirrups is shown in Figure 7a, where the behavior of specimens 20 × 30 @ 100, 20 × 30 @ 150, and 20 × 30 @ 200 is compared. No obvious difference was observed before the loading level reached 300 kN, after which the specimens were greatly damaged. Decreasing the spacing of FFRP stirrups—i.e., increasing the ratio of shear reinforcement—effectively increased the shear capacity. As the spacing of FFRP stirrups halved from 200 mm to 100 mm, the shear capacity of the three specimens in Figure 7a increased from 187 kN to 238 kN. The corresponding shear capacity contribution of FFRP stirrups almost doubled from 52 kN to 103 kN. Therefore, the change in shear capacity contribution was linear to the ratio of shear reinforcement.

As shown in Table 3: Test results, the ductility, defined as the ratio between loading-point vertical displacement at ultimate failure and steel yielding, was also substantially increased because of the decreased spacing of FFRP stirrups. The ductility increased from 3.6 to 5.5 as the spacing of the FFRP stirrup was halved from 200 mm. As all specimens failed due to the rupture of FFRP stirrups, the increased ratio of shear reinforcement decreased the stress level of FFRP stirrups, delaying the occurrence of FFRP rupture.

The effect due to the layer of FFRP stirrups is illustrated in Figure 7b,c, using the test results of specimens 10 × 30 @ 200, 20 × 30 @ 200, 10 × 60 @ 100, and 20 × 60 @ 100. The variance in the FFRP layer mostly affected the structural behavior by increasing ductility, with the shear capacity remaining almost the same. The rupture of the FFRP stirrup occurred at almost the same loading level for specimens 10 × 30 @ 200 and 20 × 30 @ 200, whose ratio of FFRP stirrups was 0.8% and 1.3%, respectively. Though increasing the layer of FFRP stirrups increased the area, the effective strength of FFRP stirrups upon FFRP rupture was decreased, which is explained in the following context.

Figure 7d shows that increasing the width of the FFRP stirrup from 30 mm to 60 mm slightly decreased the shear capacity from 306 kN (specimen 20 × 30 @ 100) to 298 kN (specimen 20 × 60 @ 100). Meanwhile, the corresponding ductility greatly increased from 5.5 to 7.7. As the spacing between FFRP stirrups was only 100 mm, the 60 mm-wide FFRP stirrups caused difficulties in vibrating concrete during specimen construction. The possible existence of voids in the concrete lowered the shear capacity.

### 3.3. Load–Strain Relation

**Figure 8:**
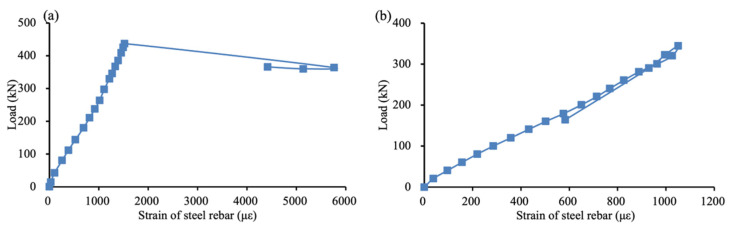
Load–strain relation of steel reinforcement bar in specimen: (**a**) 10 × 60 @ 100 and (**b**) 20 × 30 @ 150.

Another important examined structural performance is the relation between the applied load and the tensile strain of steel reinforcement bar, which is displayed in Figure 8. As the specimens were over-reinforced, steel reinforcement bar in most specimens achieved a low tensile strain. Figure 8 plots the tensile strain of the steel reinforcement bar against the applied load for specimens 10 × 60 @ 100 and 20 × 30 @ 150, reaching 6000 με and 1100 με, respectively.

**Figure 9:**
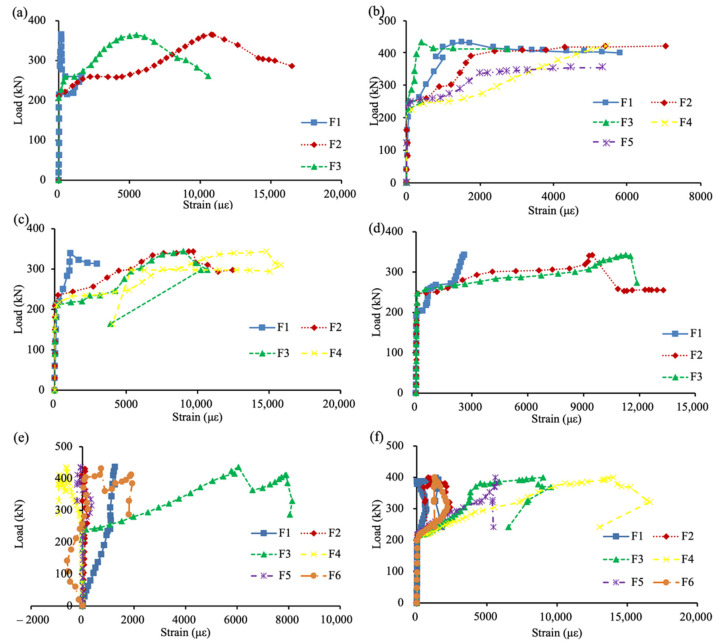
Load–strain relation of FFRP stirrups in specimen: (**a**) 10 × 30 @ 200; (**b**) 20 × 30 @ 100; (**c**) 20 × 30 @ 150; (**d**) 20 × 30 @ 200; (**e**) 10 × 60 @ 100; and (**f**) 20 × 60 @ 100.

The relation between the applied load and the tensile strain of the FFRP stirrup for all shear-reinforced specimens is shown in Figure 9. The maximum strain captured by strain gauges ranged from 8000 to 17,000 με, which are very close to the rupture strain of FFRP coupons in Figure 4. Hence, the tensile strength of FFRP stirrups was fully utilized. The low elastic modulus of FFRP yielded a low axial rigidity of FFRP stirrups. According to the strut-and-tie model, the low axial rigidity of ties—i.e., stirrups—caused a large deformation, leading to a large tensile strain of stirrups. Furthermore, the elastic modulus of the stirrup was close to that of concrete. Composite deformation between the FFRP stirrup and concrete was thus developed, which also ensured high tensile strain of the FFRP stirrup.

As for the tensile strains of FFRP stirrups in different specimens, increasing the FFRP stirrup spacing increased the respective tensile strain. For instance, as compared to specimen 20 × 30 @ 100, specimen 20 × 30 @ 200 used FFRP stirrups at a larger spacing and achieved a higher average strain in FFRP stirrups (Figure 9b,d). As can be seen from Figure 9b,f, increasing the width of FFRP stirrups also achieved similar behavior in terms of FFRP stirrup strain.

The increase in the FFRP layer decreased the tensile strain of the FFRP stirrup, which explained the similar shear capacity of specimens 10 × 30 @ 200 and 20 × 30 @ 200 (Figure 7b,c). As compared to specimen 10 × 30 @ 200 (Figure 9a), specimen 20 × 30 @ 200 (Figure 9d) used more layers of FFRP stirrups and achieved a lower tensile strain. Therefore, the tensile force applied to the specimens using more layers of FFRP stirrups was higher. The increase in tensile force and area of the FFRP stirrup eventually cancelled each other out and yielded FFRP rupture at a similar loading level. The decrease in effective strain due to the increase in the FFRP layer was similar to the concept of the reduction factor proposed in [33], where the effective strain of external carbon FRP laminates decreased with the axial rigidity of FFRP stirrups.

### 3.4. Cracking Behavior

**Figure 10:**
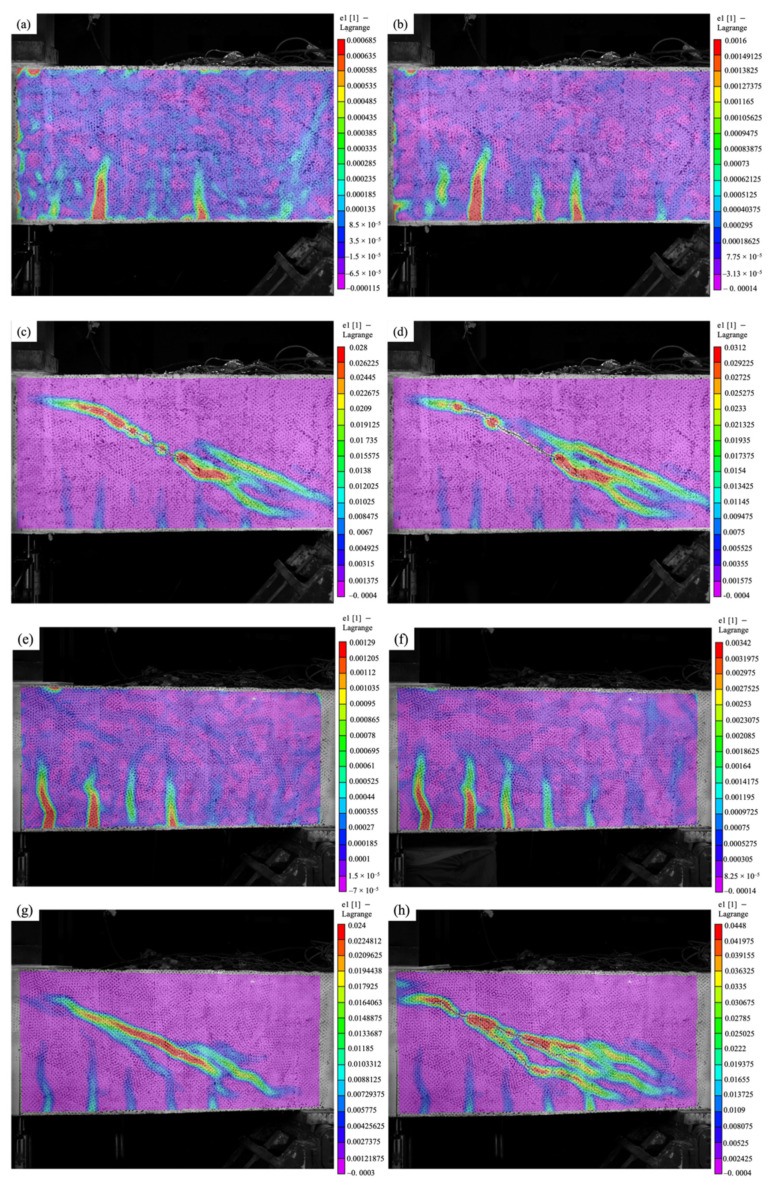
Load–strain relation of concrete for: (**a**) specimen 20 × 30 @ 200 at 0.25 *P_u_*; (**b**) specimen 20 × 30 @ 200 at 0.5 *P_u_*; (**c**) specimen 20 × 30 @ 200 at 0.75 *P_u_*; (**d**) specimen 20 × 30 @ 200 at *P_u_*; (**e**) specimen 20 × 30 @ 100 at 0.25 *P_u_*; (**f**) specimen 20 × 30 @ 100 at 0.5 *P_u_*; (**g**) specimen 20 × 30 @ 100 at 0.75 *P_u_*; and (**h**) specimen 20 × 30 @ 100 at *P_u._*

The cracking behavior of concrete monitored by the DIC system is shown in Figure 10, where specimens 20 × 30 @ 200 and 20 × 30 @ 100 are selected for illustration. The strain contours of each specimen are presented at four loading levels: 0.25 *P_u_*, 0.5 *P_u_*, 0.75 *P_u_*, and *P_u_*. Most specimens experienced several flexural cracks upon 0.25 *P_max_* and 0.5 *P_max_*, and no obvious signs of shear cracks were observed (Figure 10a,b,e,f). As the specimen approached final failure, diagonal shear cracks appeared and quickly widened (Figure 10c,d,g,h). The specimen 20 × 30 @ 200 used a FFRP stirrup of low shear reinforcement ratio, and a single major shear crack linearly connected the loading point and the support (Figure 10c,d). As the ratio of shear reinforcement increased, the crack pattern switched from a single major shear crack to multiple distributed cracks, as displayed in Figure 10g,h. In addition, flexural cracks also appeared in the tension zone of concrete, indicating that the failure mode was switched to flexural mode. For instance, specimen 10 × 60 @ 100 showed obvious signs of yielding, and the tensile strain of its steel reinforcement bar was beyond 2000 με (Figure 8a). The ultimate strain on the concrete surface also increased greatly with the ratio of shear reinforcement. For instance, the tensile strain on the concrete surface upon peak load increased from 3.1% to 4.5% (Figure 10d,h) when the ratio of shear reinforcement was doubled from specimen 20 × 30 @ 200 to 20 × 30 @ 100.

### 3.5. Crack-FFRP Strain Relation

**Figure 11:**
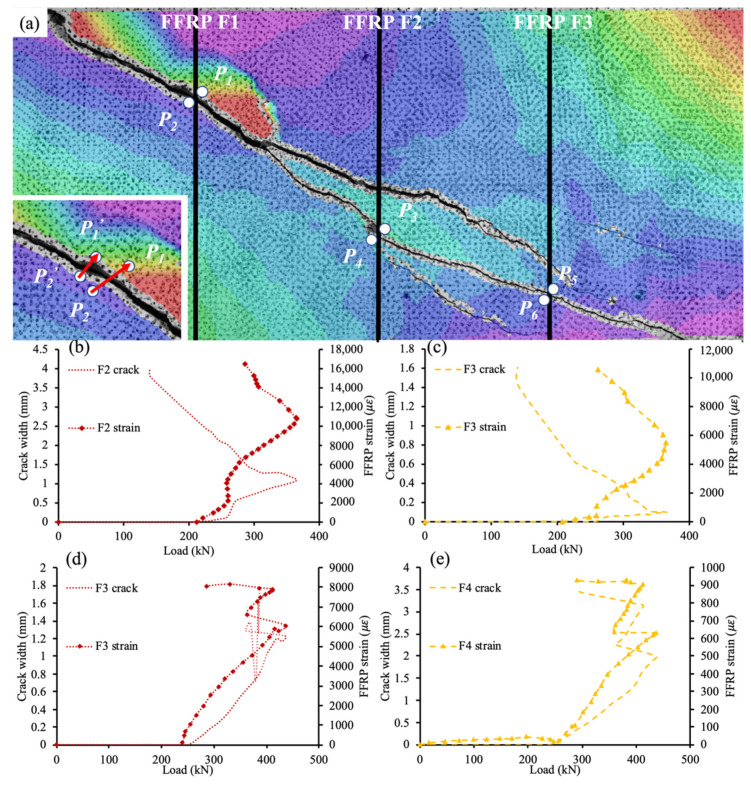
Crack-FFRP strain relation: (**a**) method; (**b**) F2 crack in specimen 10 × 30 @ 200; (**c**) F3 crack in specimen 10 × 30 @ 200; (**d**) F3 crack in specimen 10 × 60 @ 100; and (**e**) F4 crack in specimen 10 × 60 @ 100.

Figure 11 shows the relation between crack width and FFRP strain, and Figure 11a illustrates the calculating process of crack width. To determine the crack width at the intersection between the shear crack and FFRP stirrups, two points on each side of the intersection were manually selected. $p_{1}^{\prime }$, $p_{2}^{\prime }$ were two points before deformation, and *P*_1_, *P*_2_ were the same points after deformation. By tracking the vector connecting the selected points (see the enlarged picture) throughout the loading process, the crack width can be determined using the following equation:

(9) $$\delta =\frac{\bar{P_{1}P_{2}}\bar{P_{1}^{\prime }P_{2}^{\prime }}}{\left|\bar{P_{1}^{\prime }P_{2}^{\prime }}\right|}-\left|\bar{P_{1}^{\prime }P_{2}^{\prime }}\right|$$

where *P*_1_, *P*_2_ = selected points after deformation; $p_{1}^{\prime }$, $p_{2}^{\prime }$ = selected points before deformation; and *δ* = crack width. Equation (9) projects the vector after deformation to its direction before deformation, and the difference between the length of the projected vector and initial vector becomes the crack width. As the shear crack (Figure 6) almost coincided with the layout of strain gauges on FFRP stirrups (Figure 5a), the strains read from gauges were approximately treated as the strains of FFRP stirrups at the calculated crack width.

Figure 11b–e illustrate the relation between crack width and FFRP strain for four intersections on two different specimens (two intersections each specimen): specimen 10 × 30 @ 200 and specimen 10 × 60 @ 100. In each subplot, the primary vertical axis on the left side plots the crack width against load on the horizontal axis, and the secondary vertical axis on the right side plots the FFRP strain against load. All subplots follow a similar trend: the FFRP strain increased as the shear crack became wider until the peak shear capacity was reached, and the shear crack width increased monotonically after the shear capacity started decreasing. For instance, the tensile strain of the F2 stirrup (Figure 11a) reached 11,000 με upon peak load at 365 kN (Figure 11b). Meanwhile, the FFRP stirrup F1 ruptured at P1 and P2 (Figure 11a) leading to decreasing load capacity. However, the FFRP F2 continued contributing to shear resistance, represented by a further increase in FFRP strain in Figure 11b. Therefore, the shear capacity contributed by the unruptured FFRP stirrup increased monotonically throughout the loading process.

### 3.6. Shear Capacity Contribution

**Figure 12:**
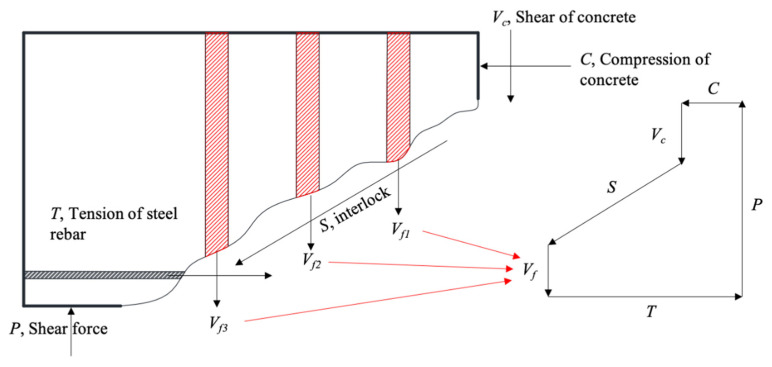
Force equilibrium.

The total shear capacity and the contributions by concrete and the FFRP stirrup are summarized in Table 4 and analyzed in the following context. Consider the force equilibrium in Figure 12; the total shear force applied by the support is resisted by multiple mechanisms: tensile force of FFRP stirrups, shear resistance provided by concrete in compression, aggregate interlocking along the shear crack, and dowel effect of steel reinforcement bar. The first term refers to the shear resistance of FFRP stirrups, and the remaining terms are categorized into the shear capacity contributed by concrete.

**Figure 13:**
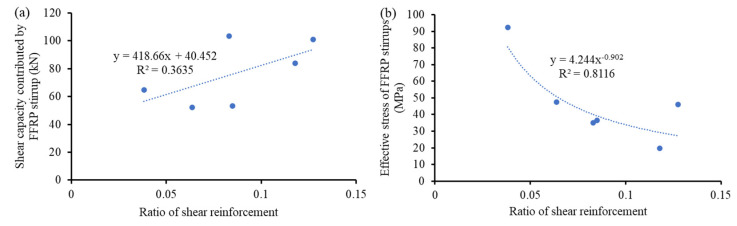
Behavior of FFRP stirrup: (**a**) shear capacity contribution and (**b**) effective stress.

The shear capacity of the reference specimen was the shear capacity contributed by concrete, which is assumed to be identical for all the other specimens using FFRP stirrups. Hence, the difference in shear capacities of the specimens using FFRP stirrups and the control specimen became the shear capacity contributed by FFRP stirrups. As shown in Figure 13a, increasing the ratio of the FFRP stirrup effectively increased the shear capacity contributed by the FFRP stirrup. Based on the obtained shear capacity contributed by the FFRP stirrup, the effective stress of the FFRP stirrup can be thus determined using Figure 12 and the following equation:

(10) $$f_{f}=\frac{V_{f}}{\sum^{\;}A_{fi}}$$

where *f_f_* = effective stress of FRP stirrup; *V_f_* = shear capacity contributed by FFRP stirrup; and *A_fi_* = area of the *i*^th^ FFRP stirrup. The calculated effective strain of FFRP stirrups is plotted against the shear reinforcement ratio in Figure 13b. Obviously, the effective stress/strain of FFRP stirrups followed a decreasing power law as the ratio of the FFRP stirrup increased, indicating a decreased efficiency of the FFRP stirrup. To achieve the optimum strengthening performance and efficiency, the shear reinforcement of the FFRP stirrup should be around 7%.

Table 4 also displays the calculated shear capacity contribution from concrete and FFRP stirrups using the following design equation from ACI 318-19 [4] and CSA S806-2012 [12], respectively:

(11) $$V_{c}=0.17\lambda \sqrt{f_{c}^{\prime }}bd$$

(12) $$V_{f}=\frac{0.4\phi_{f}A_{f}f_{f}d}{s_{f}}cot\theta$$

where *V_c_* = shear capacity contributed by concrete; λ = modification factor for lightweight concrete = 1.0; $f_{c}^{\prime }$ = concrete compressive strength; *b* = beam width; *d* = effective depth of beam; *ϕ_f_* = resistance factor for FRP = 0.75; *A_f_* = area of FRP reinforcement; *f_f_* = effective stress of FRP stirrup = *E_f_* *ϵ_f_*; *E*_f_ = Young’s modulus of FRP reinforcement; *ϵ_f_* = effective strain of FRP stirrup = 0.005; *s_f_* = spacing of FRP reinforcement; and *θ* = angle of shear crack (assumed to be 45 degrees).

The comparison between the predicted and the experimental shear capacity in Table 4 shows that shear capacity contributions were substantially underestimated by the design code. For the shear capacity contributed by FFRP particularly, the allowable effective strain was greatly lower than the experimental maximum tensile strain of FFRP stirrups (Figure 9). In consideration of the low R-square value in Figure 13, the scatters associated with the material and structural performance of FFRP stirrups were high. The current allowable strain provided a sufficient safety margin. Moreover, the shear capacity by concrete was also underestimated. This is possibly because the method proposed in Equation (11) relates the tensile strength of concrete to its shear capacity contribution, which only considers the mechanism of aggregate interlock. Other important mechanisms—such as the shear resistance of compressive concrete and the dowel effect of steel reinforcement bar—were thus ignored, leading to the underestimation of the shear capacity contribution from concrete.

## 4. Environmental Performance

The other important aspect examined in this study was the environmental performance. The life cycle assessment (LCA) approach has been widely adopted [34,35,36] to evaluate environmental performance and identify the main contributors. The LCA results provide lawmakers with a perspective from environmental performance besides the traditional structural performance. As per the international standard ISO 14040-06 [37], the life cycle assessment includes four phases: goal and scope, inventory analysis, impact assessment, and interpretation, which are elaborated in the following context.

### 4.1. Inventory Analysis and Method

The goal of this section is to assess and compare the environmental performance of different stirrups: steel, FFRP, and CFRP ones. From the assessment of structural performance, increasing the ratio of the shear reinforcement effectively improved the shear behavior, which presumably also increased the environmental impacts. To establish a fair comparison, the LCA approach assesses the efficiency of different stirrups. The functional unit refers to the material whose impacts are calculated by a life-cycle assessment (LCA), which is the stirrup needed to achieve a unit shear capacity contribution (i.e., 1 kN).

In the following context, specimen 10 × 30 @ 200 was selected to calculate the environmental performance of FFRP stirrups. The FU of FFRP stirrups in specimen 10 × 30 @ 200 is calculated by dividing the total weight of FFRP stirrups with respect to the corresponding shear capacity contribution.

The amount of steel and CFRP stirrups are calculated using the following equation from ACI 318 [4]:

(13) $$V_{s}=\frac{A_{s}f_{y}d}{s_{s}}$$

(14) $$V_{f}=\frac{A_{f}E_{f}\varepsilon_{fe}d_{f}}{s_{f}}\left(\sin\alpha_{f}+\cos\alpha_{f}\right)$$

where *V_s_* = shear capacity contributed by steel stirrup; *A_s_* = area of steel stirrup; *f_y_* = yielding strength of steel stirrup, assumed to be 400 MPa; *s_s_* = spacing of steel stirrup; *ε*_fe_ = effective strain of FRP reinforcement = min (0.75 *ε*_fu_, 0.004); *ε*_fu_ = ultimate strain of FRP shear reinforcement; and *α_f_* = orientation of FRP reinforcement with respect to horizontal axis. The shear capacities of steel and CFRP stirrups were equal to that of FFRP stirrups in specimen 10 × 30 @ 200 (i.e., 64.6 kN).

Though an equal-amount approach is often adopted to determine the FU, such an equal mass or equal volume-based approach may overestimate the ecological burden of advanced materials. For instance, UHPC is an advanced cementitious material with high strength and low permeability. To fulfill the same structural function, the amount of UHPC is significantly less than that of conventional concrete. To recognize and account for the vastly different mechanical properties and usage on the structural level, this study proposed a normalization approach, which normalizes the total environmental impacts of stirrups by the increased shear capacity due to stirrups [21,38]. The corresponding FUs are calculated and summarized in Table 5.

The major drawback of the normalization approach, however, is that this approach implicitly assumes a linear relation between the amount of material and structural behavior. For instance, doubling the number of stirrups (steel, synthetic FRP, or FFRP) in this study does not necessarily double the increase in load capacity. Furthermore, the increased shear capacity by stirrups was predicted using a design code, the conservative nature of which leads to the overestimation of environmental performance. It should also be noted that such an overestimate is identical to the considered stirrups and does not invalidate the results of the relative comparison.

**Figure 14:**
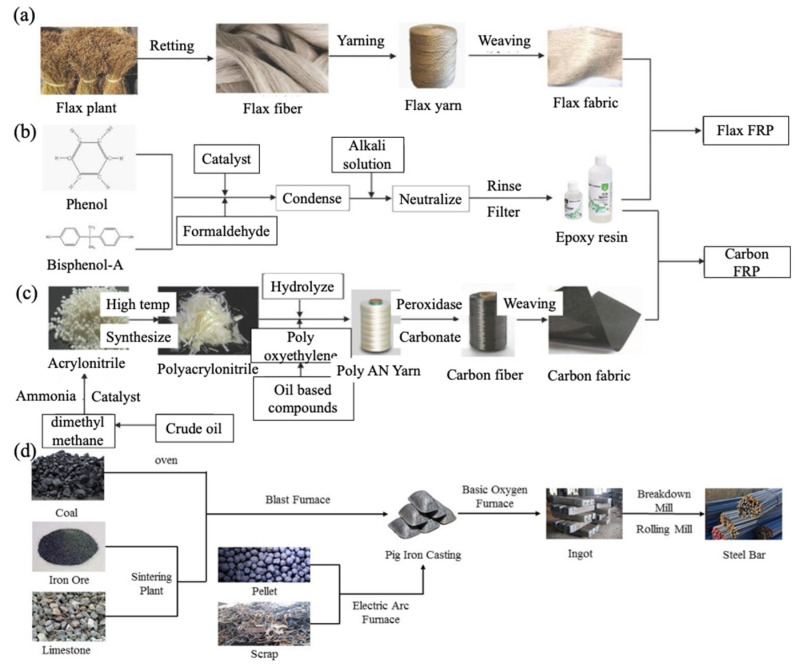
System boundary: (**a**) flax fabric; (**b**) epoxy resin; (**c**) carbon fabric; and (**d**) steel reinforcement bar.

The system boundary of the FFRP stirrup includes two paths: the path of flax fabric (Figure 14a) starts from the seeding of the flax plant, goes through the fiber retting and flax yarning, and ends at the fabric weaving; the path of epoxy resin (Figure 14b) starts from phenol and bisphenol-A, undergoes catalyst, condensation, and neutralization, and finally ends in epoxy resin. The system boundary of CFRP stirrups also has two paths: the path of epoxy resin exactly follows that in the FFRP stirrup; the path of carbon fabric (Figure 14c) originates from crude oil, which is followed by various treatment methods (such as catalyst, hydrolyzation, peroxidization, and weaving), and ends in carbon fabric. As illustrated in Figure 14d, the system boundary of the steel stirrup starts from mining and treatment of natural resources and ends in manufacturing.

Table 6 lists the life cycle inventory—or the source of background data—in the LCA; two main sources are the databases in Gabi Professional Software [39] and input data from Ecoinvent [37,40]. The Ecoinvent database was mainly used for the LCI of natural FRP, including flows of flax cultivation, flax fiber retting, jute yarning, and jute weaving. Due to the absence of data for flax yarning and flax weaving, the flows of jute yarning and jute weaving were adopted instead to represent corresponding flows of flax. The process of epoxy resin from the Gabi Professional database was adopted for the epoxy binder in carbon fiber fabric. The aggregate process of steel reinforcement bar from Gabi Extension database XXII Fraunhofer was used for the LCI of the steel stirrup in this study.

Based on the LCI of different materials, the ReCiPe midpoint method is applied in this section to evaluate the environmental performance of different stirrups. The environmental impacts in terms of (1) global warming (global warming potential (GWP)), (2) energy consumption (fossil depletion potential (FDP)), (3) human health (human toxicity potential (HTP)), (4) air quality (ozone depletion potential (ODP), particulate matter formation potential (PMFP), and photochemical oxidant formation potential (POFP)), (5) water environment (freshwater eutrophication potential (FEP) and freshwater ecotoxicity potential (FETP)), (6) marine environment (marine eutrophication potential (MEP) and marine ecotoxicity potential (METP)), and (7) terrestrial environment (terrestrial acidification potential (TAP) and terrestrial ecotoxicity potential (TETP)) are calculated and analyzed. These seven aspects are represented by a total of twelve potentials to comprehensively reflect the environmental performance. No total score is given, and any authorities and lawmakers can reach their own conclusion in a case-by-case manner.

### 4.2. Results

**Figure 15:**
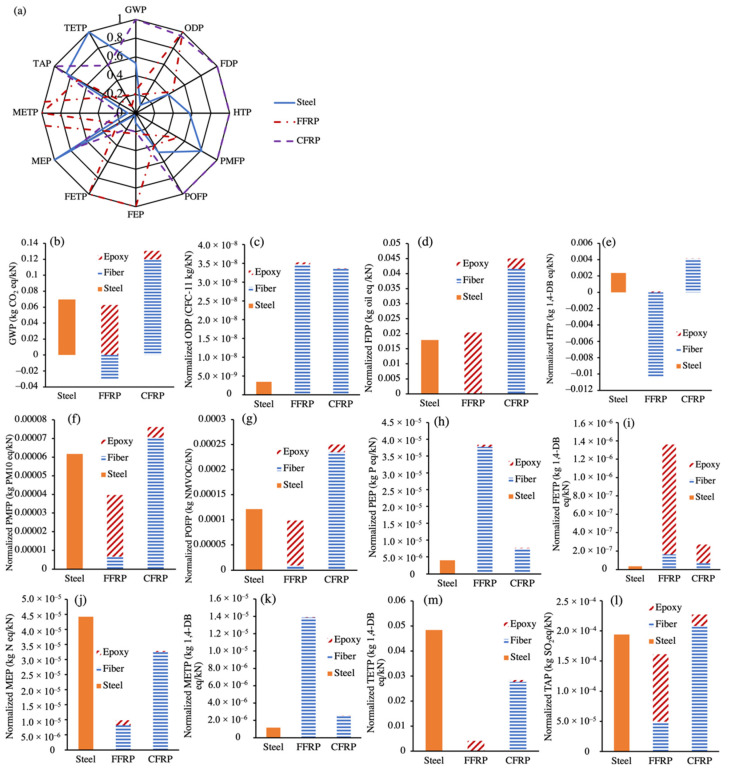
Environmental performance of steel, FFRP, and CFRP stirrups: (**a**) summary; (**b**) GWP; (**c**) ODP; (**d**) FDP; (**e**) HTP; (**f**) PMFP; (**g**) POFP; (**h**) PEP; (**i**) FETP; (**j**) MEP; (**k**) METP; (**m**) TETP ; and (**l**) TAP.

The summary of the environmental performance of steel and FFRP stirrups is illustrated in Figure 15. Environmental performance of steel, FFRP, and CFRP stirrups: (a) summary; (b) GWP; (c) ODP; (d) FDP; (e) HTP; (f) PMFP; (g) POFP; (h) PEP; (i) FETP; (j) MEP; (k) METP; (l) TAP; and (m) TETP, where each indicator is normalized by the larger potential. Each potential is presented by contributions from different materials (e.g., fiber and epoxy resin for FRP) in Figure 15b–m. Out of all considered aspects, the CFRP stirrups achieved the highest environmental impact in six potentials (GWP, FDP, HTP, PMFP, POFP, and TAP), which are related to fossil depletion, global warming, human health, and air quality. Moreover, the CFRP stirrup also had pronounced impacts on ozone depletion and marine eutrophication. In other words, CFRP stirrups are the least eco-friendly solution posing a severe danger to the ecosystem and human health. Part of the reason is due to the low efficiency of synthetic fiber. Considering the difference between the design Equations (11) and (12), the shear capacity by the FRP stirrup was lower by two factors: 0.4 and ϕf. The resistance factor of FRP (ϕf) was used to represent the low efficiency due to interfacial debonding between FRP and concrete. The constant 0.4 was applied due to the stress concentration at the bent parts of FRP stirrups. Consequently, the FU of carbon fiber became even larger than that of flax fiber despite its higher allowed effective stress (Table 5). The manufacturing process of carbon fiber was catalyzed under high temperature, and the corresponding potentials of global warming, fossil depletion, and ozone depletion were thus high. The pollutants emitted to the air and water environment also posed severe dangers to human health, represented by high HTP, PMFP, and POFP.

The steel stirrups had the highest impacts in terms of MEP and TETP, and both are related to the solid wastes generated during the manufacturing process. Other high impacts such as GWP, FDP PMFP, POFP, and TAP are caused by the depleted fossil fuels and emitted air pollutants (e.g., SO_2_ and particulate matter) of the hot rolling process. This result is contradictory to the common notion relating the steel industry to high pollution, which can be explained by the mature technique and law regulations of the manufacturing process.

The FFRP stirrups achieved the highest ODP, FEP, FETP, and METP, and its impacts of FDP, PMFP, and TAP were also high. Mixed effects from flax fiber and epoxy resin could be observed. As shown in Figure 15b, for instance, the cultivation of flax fiber consumed carbon dioxide and yielded a negative GWP, whereas the opposite result was caused by epoxy resin. A similar trend was also observed in the HTP (Figure 15e), where the heavy metals consumed by flax cultivation caused negative potentials of human health. The negative environmental performance of flax fiber was due to the heavy metal ions (nickel, arsenic, chromium, etc.) released by chemical fertilizers. The corresponding impacts on fresh water and the terrestrial environment were thus high. Generally, the FFRP stirrup had an environmental performance similar to that of the steel counterpart, and its impacts on global warming and human health were benign.

## 5. Conclusions

This paper evaluates the structural and environmental performance of closed-shape stirrups with FFRP. Test results of seven RC beams using FFRP stirrups are presented to investigate the effect due to the layer, width, and spacing of FFRP stirrups. The environmental performance is assessed using life cycle assessment based on the ReCiPe midpoint method. The environmental performance of steel, FFRP, and CFRP stirrups are analyzed and compared. The following conclusions can be made:

1. FFRP stirrups greatly increased the shear capacity and the ultimate vertical displacement by 77% and 74%, respectively.
2. The shear capacity was very sensitive to the spacing of FFRP stirrups, whereas increasing the layer and width of FFRP stirrups mainly increased ductility. It is recommended to use thick and narrow FFRP stirrups at a large spacing to achieve the best structural and environmental performance.
3. The tensile strength of closed-shape FFRP stirrups upon failure reached over 80% its rupture strength, indicating that its tensile capacity was fully utilized.
4. The effective stress of FFRP stirrups decreased to around 50% of the rupture strength as the shear reinforcement ratio of FFRP stirrups increased.
5. CFRP stirrups yielded the highest environmental impact, and the steel and FFRP stirrups had similar impacts.
6. The environmental impact of the steel stirrup was related to the consumed energy and emitted air pollutants.
7. The environmental impact of the FFRP stirrup was mainly caused by the heavy metals in the chemical fertilizer released into the terrestrial and water environment.

## Figures and Tables

**Table 1:** Specimen configuration.

Specimen	FFRP Stirrup				Steel Stirrup within Reference Span		Tensile Steel Reinforcement Bar		Compressive Steel Reinforcement Bar	
	Layer	Width (mm)	Spacing (mm)	Ratio (%) ^a^	Diameter (mm)	Spacing (mm)	Diameter (mm)	No.	Diameter (mm)	No.
Reference	- ^b^	- ^b^	- ^b^	- ^b^	12	85	28	4	12	2
10 × 30 @ 200	10	30	200	0.8						
10 × 60 @ 100	10	60	100	3.7						
20 × 30 @ 100	20	30	100	2.7						
20 × 30 @ 150	20	30	150	1.8						
20 × 30 @ 200	20	30	200	1.3						
20 × 60 @ 100	20	60	100	5.3						

^a^ Not proportional, due to variance in FFRP thickness; ^b^ Not applicable.

**Table 2:** Material properties.

Material	Diameter (mm)	Yield Strength (MPa)	Elastic Modulus (GPa)	Ultimate Strength (MPa) ^a^	Ultimate Strain (%)
Steel reinforcement bar	6	482	238	551	23.8
	12	451	192	620	- ^b^
	28	450	229	617	- ^b^
Flax fiber	- ^b^	- ^b^	30 ^c^	500 ^c^	1.7 ^c^
Flax FRP	- ^b^	- ^b^	5.8	123	1.9
Epoxy	- ^b^	- ^b^	2.4 ^c^	39.9 ^c^	- ^b^
Concrete	- ^b^	- ^b^	32.7	41.3	- ^b^

^a^ Tensile strength for steel reinforcement bar, flax fiber, flax FRP, and epoxy, and compressive strength for concrete; ^b^ Not applicable; ^c^ From manufacturer.

**Table 3:** Test results.

Specimen	Yielding State		Peak State		Ultimate State			
	Load (kN)	Vertical Displacement (mm)	Load (kN)	Vertical Displacement (mm)	Load (kN)	Vertical Displacement (mm)	Ductility	Angle of Shear Crack (°) ^a^
Reference	214	2.6	247	4.0	195	6.6	2.6	27.7
10 × 30 @ 200	211	1.9	365	4.2	283	5.0	2.7	25.4
10 × 60 @ 100	232	2.2	437	5.8	383	11.5	5.3	23.4
20 × 30 @ 100	210	2.0	432	6.9	306	11.2	5.5	25.8
20 × 30 @ 150	220	2.0	344	5.8	298	7.9	3.9	30.3
20 × 30 @ 200	215	1.8	343	4.4	218	6.6	3.6	27.1
20 × 60 @ 100	205	1.9	401	12.3	320	14.3	7.7	26.2

^a^ Represented by the major shear crack.

**Table 4:** Shear capacity contribution.

Specimen	Shear Capacity (kN)	Experimental		Predicted	
		*V_c_* (kN)	*V_f_* (kN)	*V_c_* (kN)	*V_f_* (kN)
Control	135	135	- ^a^	49	- ^a^
10 × 30 @ 200	199		64.6		8.3
10 × 60 @ 100	238		103.4		33.1
20 × 30 @ 100	236		101.1		33.8
20 × 30 @ 150	188		53.1		22.5
20 × 30 @ 200	187		52.2		16.9
20 × 60 @ 100	219		84.0		67.5

^a^ Not applicable.

**Table 5:** Functional unit.

Functional Unit (g/kN)	Stirrup		
	CFRP	FFRP	Steel
Fiber	3.4	2.7	- ^a^
Epoxy resin	1.1	6.7	- ^a^
Steel	- ^a^	- ^a^	30.3

^a^ Not applicable.

**Table 6:** Life cycle inventory.

Material	Source	
	Flow	Database
Flax fiber	flax production market for flax plant flax retting, market for flax fiber yarn production market for yarn textile production market for textile	Ecoinvent 3.6 database [40]
Steel reinforcement bar	Asia: Steel rebar	Gabi professional database [39]
Epoxy resin	Epoxy resin	Gabi professional database [39]
Carbon fiber	Carbon fiber (CF; PAN-based; HT) Carbon fiber fabric (250 g/m^2^; not bindered)	Gabi Extension database XXII Fraunhofer [39]

## Data Availability

Data available on request due to restrictions, e.g., privacy or ethical. The data presented in this study are available on request from the corresponding author.

## References

[1] Sae-Long W., Limkatanyu S., Panedpojaman P., Prachasaree W., Damrongwiriyanupap N., Kwon M., Hansapinyo C. (2021). Nonlinear Winkler-based Frame Element with Inclusion of Shear-Flexure Interaction Effect for Analysis of Non-Ductile RC Members on Foundation. J. Appl. Comput. Mech..

[2] Lam L., Teng J.G. (2003). Design-oriented stress-strain model for FRP-confined concrete. Constr. Build. Mater..

[3] Dong Z.Q., Wu G., Xu B., Wang X., Taerwe L. (2016). Bond durability of BFRP bars embedded in concrete under seawater conditions and the long-term bond strength prediction. Mater. Des..

[4] (2019). Building Code Requirements for Structural Concrete and Commentary.

[5] (2014). Design of concrete structures.

[6] Cao S.Y., Chen J.F., Teng J., Hao Z., Chen J. (2005). Debonding in RC beams shear strengthened with complete FRP wraps. J. Compos. Constr..

[7] Harries K.A., Aidoo J. (2006). Debonding- and fatigue-related strain limits for externally bonded FRP. J. Compos. Constr..

[8] Chen C., Wang X.W., Sui L.L., Xing F., Chen X.L., Zhou Y.W. (2019). Influence of FRP Thickness and Confining Effect on Flexural Performance of HB-Strengthened RC Beams. Compos. Part B Eng..

[9] Kalfat R., Al-Mahaidi R., Smith S.T. (2013). Anchorage Devices Used to Improve the Performance of Reinforced Concrete Beams Retrofitted with FRP Composites: State-of-the-Art Review. J. Compos. Constr..

[10] Wu Y.F., Huang Y. (2008). Hybrid bonding of FRP to reinforced concrete structures. J. Compos. Constr..

[11] (2017). Guide for the Design and Construction of Externally Bonded FRP Systems for Strengthening Concrete structures.

[12] (2012). Design and Construction of Building Components with Fiber-Reinforced Polymer.

[13] (2001). Fib Task Group, Externally Bonded FRP Reinforcement for RC Structures, 9.3.

[14] Jena T., Kaewunruen S. (2021). Life Cycle Sustainability Assessments of an Innovative FRP Composite Footbridge. Sustainability.

[15] Chen J., Chouw N. (2018). Flexural behaviour of flax FRP double tube confined coconut fibre reinforced concrete beams with interlocking interface. Compos. Struct..

[16] Yan L., Chouw N., Jayaraman K. (2014). Flax fibre and its composites—A review. Compos. Part B Eng..

[17] Di Luccio G., Michel L., Ferrier E., Martinelli E. (2017). Seismic retrofitting of RC walls externally strengthened by flax-FRP strips. Compos. Part B Eng..

[18] Yan L., Su S., Chouw N. (2015). Microstructure, flexural properties and durability of coir fibre reinforced concrete beams externally strengthened with flax FRP composites. Compos. Part B Eng..

[19] Chen C., Yang Y., Yu J., Yu J., Tan H., Sui L.L., Zhou Y.W. (2020). Eco-friendly and mechanically reliable alternative to synthetic FRP in externally bonded strengthening of RC beams: Natural FRP. Compos. Struct..

[20] Huang L., Yan B., Yan L., Tan H. (2016). Reinforced concrete beams strengthened with externally bonded natural flax FRP plates. Compos. Part B Eng..

[21] Chen C., Yang Y., Zhou Y., Xue C., Chen X., Wu H., Sui L.L., Li X. (2020). Comparative analysis of natural fiber reinforced polymer and carbon fiber reinforced polymer in strengthening of reinforced concrete beams. J. Clean. Prod..

[22] Gu F., Zheng Y., Zhang W., Yao X., Pan D., Wong A.S.M., Guo J., Hall P., Sharmin N. (2018). Can bamboo fibres be an alternative to flax fibres as materials for plastic reinforcement? A comparative life cycle study on polypropylene/flax/bamboo laminates. Ind. Crops Prod..

[23] Luo G., Li X., Zhou Y., Sui L., Chen C. (2021). Replacing steel stirrups with natural fiber reinforced polymer stirrups in reinforced concrete Beam: Structural and environmental performance. Constr. Build. Mater..

[24] Tahir M., Wang Z., Ali K.M. (2019). Axial compressive behavior of square concrete columns confined with CFRP strip ties using wet lay-up technique. Constr. Build. Mater..

[25] Lee C., Ko M., Lee Y. (2014). Bend strength of complete closed-type carbon fiber reinforced polymer stirrups with rectangular section. J. Compos. Constr..

[26] Lee C., Lee S., Shin S. (2015). Shear capacity of RC beams with carbon fiber-reinforced polymer stirrups with rectangular section. J. Compos. Constr..

[27] Sen T., Jagannatha Reddy H.N. (2014). Efficacy of bio derived jute FRP composite based technique for shear strength retrofitting of reinforced concrete beams and its comparative analysis with carbon and glass FRP shear retrofitting schemes. Sustain. Cities Soc..

[28] International Organization for Standard (ISO) (1997). Plastics—Determination of Tensile Properties—Part 4: Test Conditions for Isotropic and Orthotropic Fibre-Reinforced Plastic Composites.

[29] (2010). Code for Design of Concrete Structures.

[30] (2012). Standard Test Method for Concrete Structures.

[31] (2019). Standard Specification for Deformed and Plain Stainless Steel Bars for Concrete Reinforcement.

[32] Feng P., Cheng S., Bai Y., Ye L.P. (2015). Mechanical behavior of concrete-filled square steel tube with FRP-confined concrete core subjected to axial compression. Compos. Struct..

[33] Khalifa A., Gold W.J., Nanni A., Aziz A. (1998). Contribution of externally bonded FRP to shear capacity of RC flexural members. J. Compos. Constr..

[34] Ben-Alon L., Loftness V., Harries K.A., DiPietro G., Hameena E.C. (2019). Cradle to site Life Cycle Assessment (LCA) of natural vs conventional building materials: A case study on cob earthen material. Build. Environ..

[35] Quintana A., Alba J., del Rey R., Guillen-Guillamon I. (2018). Comparative Life Cycle Assessment of gypsum plasterboard and a new kind of bio-based epoxy composite containing different natural fibers. J. Clean. Prod..

[36] Zhou C., Shi S.Q., Chen Z., Cai L., Smith L. (2018). Comparative environmental life cycle assessment of fiber reinforced cement panel between kenaf and glass fibers. J. Clean. Prod..

[37] (2006). Environmental Management—Life Cycle Assessment—Principles and Framework.

[38] Jirawattanasomkul T., Minakawa H., Likitlersuang S., Ueda T., Dai J.-G., Wuttiwannasak N., Kongwang N. (2021). Use of water hyacinth waste to produce fibre-reinforced polymer composites for concrete confinment: Mechanical performance and environmental assessment. J. Clean. Prod..

[39] (2012). GaBi: Software and Database Contents for Life Cycle Engineering.

[40] Wernet G., Bauer C., Steubing B., Reinhard J., Moreno-Ruiz E., Weidema B. (2016). The ecoinvent database version 3 (part I): Overview and methodology. Int. J. Life Cycle Assess..

